# Pediatric Thyroid Cancer Multidisciplinary Team Improves Adherence of Radioactive Iodine Treatment According to National Guidelines

**DOI:** 10.1002/ohn.1376

**Published:** 2025-08-08

**Authors:** Shravan Gowrishankar, Dylan Thompson, Sara K. Bartz, Lindsey S. Johnstone, Kalpnaben Patel, Chiraysu Shah, Sara H. Duffus, Ryan H. Belcher

**Affiliations:** ^1^ Vanderbilt Department of Otolaryngology–Head and Neck Surgery Nashville Tennessee USA; ^2^ Division of Pediatric Endocrinology Monroe Carell Jr. Children's Hospital at Vanderbilt Nashville Tennessee USA; ^3^ Department of Radiology Vanderbilt University Medical Center Nashville Tennessee USA; ^4^ Department of Nuclear Medicine Vanderbilt University Medical Center Nashville Tennessee USA; ^5^ Division of Pediatric Endocrinology University of North Carolina at Chapel Hill Chapel Hill North Carolina USA; ^6^ Division of Pediatric Otolaryngology–Head and Neck Surgery Monroe Carell Jr. Children's Hospital at Vanderbilt Nashville Tennessee USA

**Keywords:** ATA 2015 guidelines, MDT, multidisciplinary team, pediatric, pediatric thyroid guidelines, radioactive iodine, RAI, thyroid cancer

## Abstract

**Objective:**

To evaluate whether a multidisciplinary team (MDT) approach improves adherence to the 2015 American Thyroid Association (ATA) postoperative surveillance guidelines in pediatric thyroid cancer patients.

**Methods:**

A retrospective review at our institution was performed on pediatric patients who had thyroidectomy for papillary or follicular thyroid cancer between 2017 and 2024. Patients who were treated from 2017 to 2020 (pre‐MDT) were compared to those treated from 2021 to 2024 (post‐MDT), following the establishment of the MDT in January 2021. Data collected included timing of postoperative thyroglobulin (Tg) tests, iodine‐123 (^123^I) diagnostic scans, iodine‐131 (^131^I) administration, and the number of endocrinologists involved in care.

**Results:**

From 2017 to 2024, 47 patients were identified (n = 28 from 2017 to 2020; n = 19 from 2021 to 2024). Median age was 15 years. ^131^I was administered in 47% of low‐risk patients from 2017 to 2020 (n = 7/15) and in 0% of low‐risk patients from 2021 to 2024 (n = 0/7) (*P* = .04) based on ATA guidelines. Between the two time periods, there was an increased percentage of patients who had postoperative Tg tests or ^123^I diagnostic scans according to the ATA guidelines.

**Discussion:**

There were significantly fewer low‐risk patients who received ^131^I treatment in the 2021 to 2024 group compared to the 2017 to 2020 group. A greater proportion of patients received guideline‐recommended postoperative test in the second time period. Introduction of the MDT and fewer endocrinologists involved in postoperative care of each patient from 2021 is associated with improved adherence to the ATA guidelines for pediatric thyroid cancer patients.

**Implications for Practice:**

Our study supports the importance of the MDT in the postoperative care of pediatric thyroid cancer patients.

Until 2015, postoperative management of pediatric thyroid cancer patients was based on published adult protocols given a lack of pediatric‐specific guidelines.^1^, ^2^ This required all children to undergo radioiodine treatment with iodine‐131 (^131^I) following surgery. While this was associated with high cure rates initially, follow‐up studies over time revealed an increased mortality risk in these children due to additional malignancies arising in those possibly overtreated with radioactive iodine.^3^, ^4^ Consequently, this prompted several national agencies to publish separate guidelines for the postoperative management of pediatric thyroid cancer patients.^5^, ^6^ In the United States, the most recent pediatric guidelines were published by the American Thyroid Association (ATA) in 2015.^5^ These guidelines stratified pediatric patients into low‐, intermediate‐, and high‐risk based on the extent of local disease and regional lymph node status using the tumor‐node‐metastasis (TNM) system.^7^ Those with cancer confined to the thyroid with N0/NX disease are classified as low‐risk; those with extensive N1a or minimal N1b disease are classified as intermediate‐risk; and those with invasive disease (T4) or regionally extensive disease (extensive N1b) are classified as high‐risk.^5^

According to ATA guidelines, the initial postoperative assessment for low‐risk patients involves measuring unstimulated thyroglobulin (Tg), whereas for intermediate‐ and high‐risk patients, it involves measuring thyroid‐stimulating hormone (TSH)‐stimulated Tg and diagnostic iodine‐123 (^123^I) scanning. RAI administration is recommended in those deemed intermediate‐ and high‐risk, limiting overtreatment in low‐risk children unlikely to benefit. This is supported by recent evidence showing that withholding ^131^I treatment in low‐risk children does not negatively affect remission rates.^8^, ^9^

To improve overall care in line with these guidelines, the ATA pediatric thyroid guidelines also recommends including a multidisciplinary team (MDT) in the care of pediatric thyroid cancer patients.^5^ Discussing each patient's postoperative care in an MDT consisting of surgeons, endocrinologists, pathologists, radiologists, oncologists, nuclear medicine specialists, and nurse coordinators allows interdisciplinary decisions on optimal therapy and follow‐up.^10^ At our center, an MDT involving the above specialists was started in January 2021. In this study, we evaluate how our institution's pediatric thyroid cancer patients were managed according to the ATA 2015 guidelines before and after the introduction of this MDT.

## Methods

Pediatric patients (<18 years at time of surgery) who underwent total thyroidectomy or completion thyroidectomy for thyroid cancer at Monroe Carell Jr. Children's Hospital at Vanderbilt between 2017 and 2024 were included in this study. This study was approved by the Institutional Review Board of Vanderbilt University Medical Center (IRB Number: 241972). The time period included 4 years before (2017‐2020) and 4 years after (2021‐2024) the introduction of the MDT in January 2021.

Retrospective review of patient's charts were analyzed for the following: age at surgery; gender; date of thyroidectomy; postoperative risk status according to ATA guidelines^5^; subtype of thyroid cancer on pathology; timing of Tg and TSH‐stimulated Tg tests performed after surgery; timing and dose of postoperative ^123^I for diagnostic scanning; timing and dose of postoperative ^131^I administration; and the number of endocrinologist physicians involved in care.

Descriptive statistics including median, interquartile range (IQR), and range were used to compare age, test timings, and ^131^I doses between groups. Fisher's exact tests were used to compare ATA risk status; gender; cancer type; proportion of patients who received Tg, TSH‐Tg; ^123^I scans; and ^131^I treatment according to ATA guidelines between 2017 to 2020 and 2021 to 2024. *t* tests were used to compare age at surgery; the timing of each test following surgery; the dose of ^123^I and ^131^I given; and the number of endocrinologist physicians involved in care in the first year following surgery between the two time groups. A *P* value < .05 was taken as the threshold for statistical significance. All statistical analyses were conducted using GraphPad Prism Version 10 for Windows.

The study employed a retrospective design, analyzing preexisting clinical data from two defined time periods. It did not involve prospective monitoring or active modifications to clinical processes during the study period. In this context, the implementation of the MDT approach occurred independently of the team conducting this particular study, and our study sought to observe its impact in a retrospective manner rather than driving or evaluating this intervention in real‐time.

## Results

### Patient Demographics

A total of 47 patients underwent thyroidectomy for thyroid cancer from 2017 to 2024 (n = 28 from 2017 to 2020; n = 19 from 2021 to 2024). Overall, median age at surgery was 15 years [IQR: 4 years; range 13 years). There were 38 female and 9 male patients. By histology, there were 46 papillary cell carcinoma cases and 2 follicular cell carcinoma cases. Breakdown of characteristics by year groups, including ATA risk status, is given in Table 1.

**Table 1:** Patient Demographics and Characteristics by Year Groups

	2017‐2020 (n = 28)	2021‐2024 (n = 19)	*P* value
ATA risk status			
Low	11	6	.76
Intermediate	3	5	.24
High	14	8	.77
Median age in years (IQR) [range]	15 (3) [12]	15 (4) [13]	.46
Gender			
Female	23	15	1.0
Cancer type			
PTC	27	18	1.0
FTC	1	1	1.0

Abbreviations: ATA, American Thyroid Association; FTC, follicular thyroid cancer; IQR, interquartile range; PTC, papillary thyroid cancer.

### Postoperative Tg Testing

Unstimulated Tg testing was performed in 23 patients from 2017 to 2020 and in 12 patients from 2021 to 2024. This was indicated in 11 patients from 2017 to 2020 and in 6 patients from 2021 to 2024 (ie, low‐risk patients). Unstimulated postoperative Tg was performed in 91% of patients where it was indicated as an initial test from 2017 to 2020 (n = 10/11), and in 100% of those indicated as an initial test from 2021 to 2024 (n = 6/6) (*P* = 1.0). Unstimulated postoperative Tg was performed in 76% of patients where it was not indicated from 2017 to 2020 (n = 13/17) and in 46% of patients where it was not indicated from 2021 to 2024 (n = 6/13) (*P* = .13).

**Figure 1:**
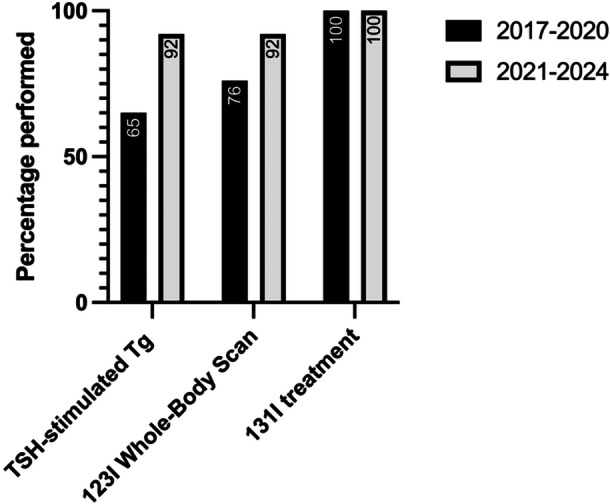
Changes in the performance of thyroid‐stimulating hormone (TSH)‐stimulated thyroglobulin (Tg) testing, ^123^Iodine whole‐body scans, and ^131^Iodine treatment in those indicated according to American Thyroid Association guidelines across the two time periods. ^123^I, iodine‐123; ^131^I, iodine‐131.

Postoperative TSH‐stimulated Tg was performed in 14 patients from 2017 to 2020 and in 14 patients from 2021 to 2024. This test was indicated in 17 patients from 2017 to 2020 and in 13 patients from 2021 to 2024 (ie, intermediate and high‐risk patients). Stimulated postoperative Tg was performed in 65% of patients where it was indicated from 2017 to 2020 (n = 11/17), and in 92% of indicated patients from 2021 to 2024 (n = 12/13) (*P* = .1) (Figure 1). TSH‐stimulated Tg was performed in 27% of patients where it was not indicated from 2017 to 2020 (n = 3/11), and in 33% of patients where it was not indicated from 2021 to 2024 (n = 2/6) (*P* = 1.0).

The mean time from surgery to unstimulated Tg testing in the initial time group was 136 days and 76 days in the latter group (*P* = .9). The mean time from surgery to stimulated Tg testing in the initial time group was 250 days and 120 days in the latter group (*P* = .3).

### ^123^I Diagnostic Whole‐Body Scan

Postoperative ^123^I scans were performed in 15 patients from 2017 to 2020 and in 13 patients from 2021 to 2024. According to ATA guidelines, these scans were indicated in 17 patients from 2017 to 2020 and in 13 patients from 2021 to 2024. Postoperative ^123^I scans were performed in 76% of patients where it was indicated (n = 13/17) from 2017 to 2020. In contrast, ^123^I scans were performed in 92% of indicated patients (n = 12/13) from 2021 to 2024 (*P* = .4) (Figure 1). Postoperative ^123^I scans were performed in 17% of patients where it was not indicated based on the ATA guidelines (ie, low risk with normal postoperative Tg) in both 2017 to 2020 (n = 2/12) and 2021 to 2024 (n = 1/6) (*P* = 1.0).

The mean time of ^123^I scanning from surgery was 207 days from 2017 to 2020 and 153 days from 2021 to 2024 (*P* = .5). The dose of ^123^I given in the first group was 2.7 and 2.6 mCi in the latter group (*P* = .9).

### ^131^I Treatment

**Figure 2:**
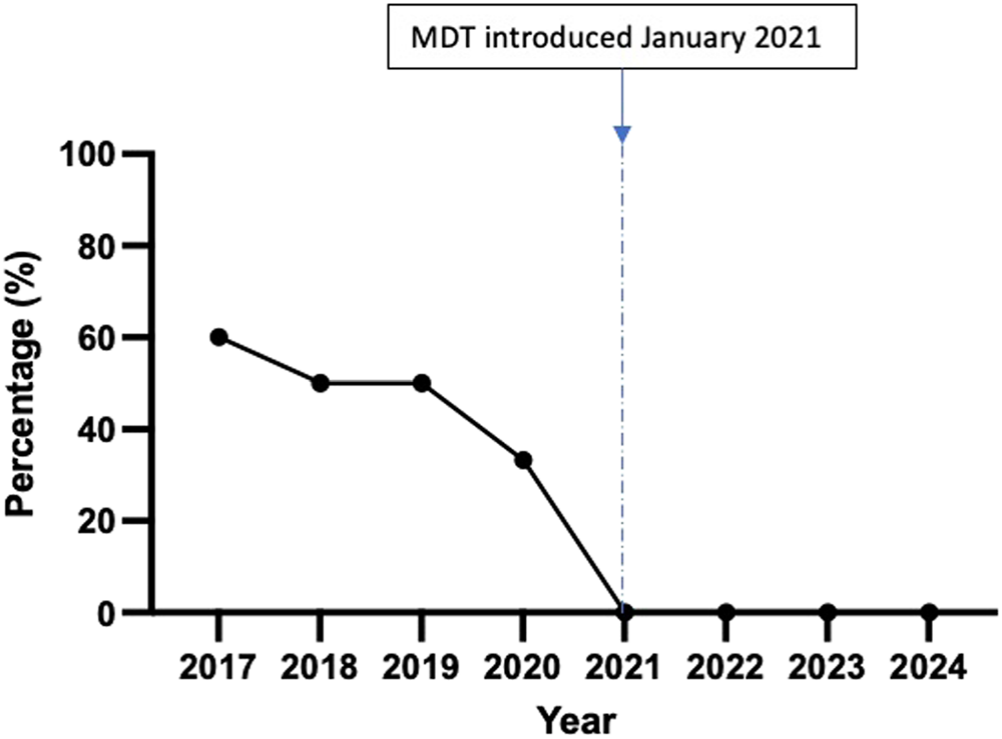
The percentage of low‐risk patients receiving iodine‐131 treatment before and after the introduction of the multidisciplinary team (MDT).

Postoperative ^131^I treatment was performed in 20 patients from 2017 to 2020 and 12 patients from 2021 to 2024. According to ATA guidelines, ^131^I treatment was indicated in 13 patients from 2017 to 2020 and 12 patients from 2021 to 2024. Postoperative ^131^I treatment was performed in 100% of patients where it was indicated in both groups (n = 13/13, and n = 12/12, respectively) (*P* = 1.0) (Figure 1). Postoperative ^131^I treatment was performed in 47% of patients where it was not indicated in the first time period (n = 7/15), and in 0% of patients where it was non‐indicated in the second time period (n = 0/7) (*P* = .04) (Figure 2). According to ATA guidelines, the reasons for non‐indication during the first time period included a clear ^123^I scan (n = 5) and proceeding directly to ^131^I treatment before an initial ^123^I diagnostic scan to assess residual disease (n = 2). At the time of last follow‐up (median: 1735 days, IQR: 1643 days; range 2314 days), none of the patients from 2017 to 2020 who received ^131^I against ATA guidelines had evidence of recurrence or secondary primaries.

The percentage of patients who received ^131^I who were high‐risk was 70% (n = 14/20) in 2017 to 2020 and 58% (n = 7/12) in 2021 to 2024 (*P* = .7). The mean time from thyroidectomy to the ^131^I treatment was 128 days from 2017 to 2020 and 203 days from 2021 to 2024 (*P* = .1). The mean dose of ^131^I given in the first group was 115.1 and 101.2 mCi in the second group (*P* = .4).

### ^131^I Treatment in Those With a Clear ^123^I Scan

From 2017 to 2020, postoperative ^123^I diagnostic scanning was performed in 15 patients, and there was no uptake in 6 of these patients. Of these six patients, four were classified as high‐risk, one was classified as intermediate‐risk, and one was classified as low‐risk. All 6 patients had postoperative TSH‐stimulated Tg values within the normal range, and all 6 patients received ^131^I treatment. From 2021 to 2024, ^123^I scans were performed in 13 patients, and there was no uptake in 2 of these patients. Of these two patients, one was intermediate‐risk and one was high‐risk. Neither of these two patients received ^131^I ablation, and both had postoperative TSH‐stimulated Tg values within the normal range. Through monitoring and surveillance, both patients have remained disease‐free without need for revision surgery or further ^131^I treatment at the time of last follow‐up (594 and 398 days from surgery, respectively).

### Number of Endocrinologists Involved in Postoperative Care

The mean number of endocrinology attending physicians (ie, MDs) involved in care per patient in the first year following surgery was 2.3 in the first group and 1.4 in the latter group (*P* = .02).

## Discussion

Following the introduction of the pediatric thyroid MDT team in 2021, we report a significantly lower percentage of low‐risk patients who received ^131^I treatment. We also note an increase in the percentage of patients who had postoperative Tg and ^123^I scans performed in accordance with ATA guidelines, although this was not statistically significant. Furthermore, we also report a significant reduction in the number of endocrinologists involved in the postoperative care of each patient from 2021 to 2024.

MDT conferences form an important part of the management of cancer patients and improve outcomes in many cancer types, including adult thyroid cancer.^11^, ^12^, ^13^, ^14^ The combined presence of experts from different medical and surgical specialties facilitates discussion on optimal management strategies in line with current treatment guidelines.^10^ Shared input in this setting can also improve treatment coordination and standardize the decision‐making process, reducing errors and variations in practice based on individual preference.^10^, ^15^, ^16^

Until the past decade, most children received ^131^I treatment following thyroid cancer surgery in line with adult guidelines.^5^ However, follow‐up studies in these children over decades revealed an increased rate of secondary malignancies attributed to radiation exposure.^3^, ^4^, ^17^ Pediatric‐specific ATA guidelines in 2015 aim to minimize radiation in low‐risk patients.^5^ These low‐risk patients included those with cancer confined to the thyroid gland with N0 disease and low postoperative Tg levels.^5^ Our findings revealed a reduction in the percentage of low‐risk patients who received ^131^I treatment from 2021 to 2024 compared to 2017 to 2020. Our institution introduced a pediatric thyroid cancer MDT in January 2021 to facilitate interdisciplinary decisions on postoperative care, which is recommended by the 2015 pediatric ATA guidelines for those treating children with pediatric thyroid cancer.^5^ Our institution's MDT includes pediatric thyroid surgeons, pediatric endocrinologists, pathologists, cytopathologists, pediatric oncologists, pediatric radiologists, and nuclear medicine radiologists who discuss each patient in a monthly tumor board conference to optimize postoperative surveillance and treatment. This allows interdisciplinary input in identifying patients who are likely to benefit from ^131^I treatment and excluding those who are unlikely to benefit, based on the pediatric ATA guidelines and the most updated literature. Discussing the ATA risk status of each patient has been standardized into the structure of the MDT conferences and likely contributed to the reduction of low‐risk patients receiving ^131^I treatment.

We also report a reduction in patients with a ^123^I whole‐body scan that reveals no or only minimal thyroid bed uptake who go on to receive ^131^I treatment after MDT implementation. Following thyroidectomy, ^123^I is used as a tracer to identify remnant cancer in the thyroid bed and distant metastases.^18^ It delivers a lower radiation dose compared to ^131^I, making it safer for diagnostic purposes in children.^19^, ^20^, ^21^ In addition, there is evidence that even a small diagnostic dose of ^131^I can “stun” thyroid cells and interfere with later ^131^I uptake and efficacy of subsequent RAI treatment.^22^, ^23^ As a result, the ATA recommends using ^123^I to detect remnant disease in intermediate and high‐risk children,^5^ and this isotope is commonly used in preference to ^131^I for this purpose in children.^21^ Those with a clear ^123^I whole‐body scan have a very low‐risk of persistent disease and are not routinely recommended to undergo ^131^I treatment.^5^ A key component of MDT meetings involves discussion of ^123^I scan results, and this could have contributed to the improved parameters noted in this study.

From 2021, we also note an improvement in the percentage of indicated patients who had unstimulated Tg, TSH‐stimulated Tg, and ^123^I diagnostic whole‐body scans according to ATA guidelines. Furthermore, we also report a reduction in the percentage of the above tests performed in those not indicated. Consequently, fewer patients receive radioactive iodine in the form of ^123^I and ^131^I where it is not indicated, minimizing radiation exposure and its associated risks such as an increased rate of future malignancies and cerebrovascular disease.^3^, ^4^, ^17^, ^24^, ^25^ This also reduces healthcare expenditure on tests that might not be necessary, reducing costs and improving resource allocation. Again, discussing indications for the above tests based on ATA risk status is a core component of MDT discussions and likely contributed to these findings. While we note improvements, we did not achieve statistical significance in the percentage of indicated patients who had unstimulated Tg, TSH‐stimulated Tg, and ^123^I diagnostic whole‐body scan, which is likely due to insufficient statistical power due to small sample sizes. Furthermore, we also note a reduction in the time from surgery to the performance of the appropriate postoperative test (ie, Tg, TSH‐stimulated Tg and ^123^I) from 2021, although not statistically significant. Timely postoperative staging is important to allow detection and subsequent treatment of residual disease if present.^26^ The ATA recommends that this is completed within 12 weeks from surgery for pediatric patients.^5^ Delays in postoperative staging can lead to remnant disease persisting for a longer time following surgery and delays to subsequent treatment including ^131^I treatment, which can increase the risk of persistent disease after treatment.^27^ The MDT meeting provides a platform for scheduling postoperative tests based on each patient's ATA risk status and overall clinical picture.^28^ At our center, the timing of each test is routinely discussed and scheduled in MDT meetings.

We also note a significant reduction in the mean number of endocrinologists involved in the postoperative care of each patient in the first year following surgery from 2021 to 2023 (n = 2.3) compared to 2017 to 2020 (n = 1.4) (*P* = .02). We chose to focus on the first year following surgery as (i) this covers the period where the majority of initial postoperative testing is done as well as ^131^I treatment if indicated, and (ii) patients from earlier time points have a greater chance of having an increased number of endocrinologists involved solely based on longer follow‐up, which introduces a source of bias if this is compared with patients from latter timepoints (eg, 2023). This study shows that an MDT may be associated with fewer endocrinologists. Having fewer endocrinologists managing and seeing these patients may improve adherence to guidelines, as those involved in a dedicated MDT are likely to be more up‐to‐date and familiar with the 2015 ATA pediatric thyroid guidelines. Evidence from other specialties suggests that having fewer points of contact when a large MDT is involved can streamline patient care and allow more efficient liaison with other health professionals when needed, improving patient outcomes.^29^, ^30^, ^31^

Our study provides data on the impact of the MDT in the postoperative care of pediatric thyroid cancer patients, a rarely studied cohort. We outline increases in the percentage of several postoperative tests and treatments performed in line with ATA guidelines following the introduction of the MDT, including ^123^I diagnostic scans and ^131^I treatment. However, our study also has limitations. This is a single‐center retrospective study, which affects generalizability because our findings reflect the specific patient population, clinical practices, and institutional protocols of our hospital, which may not capture the variability seen in different healthcare environments. Due to small sample sizes, we were unable to show statistically significant differences in some arms despite sizable changes. We also do not report on patient outcomes such as recurrence or survival, given the high 30‐year survival rate for papillary thyroid cancer in pediatric patients^32^, ^33^ and the recency and limited time scope for this study. While treatment guidelines are important in facilitating evidence‐based care, they are not prescriptive, and variation is sometimes necessary based on individual patient factors. As a result, adherence to guidelines alone does not necessarily signal a successful MDT practice. Deviation from these guidelines, as found in some cases here, might be clinically warranted. It is here that the shared expertise and experience afforded by an MDT can be particularly useful.

## Implications for Practice

Following the introduction of the pediatric thyroid MDT in 2021, we report a significant decrease in low‐risk patients being treated with ^131^I therapy in line with ATA guidelines. We also report improvements in the percentage of postoperative Tg tests and ^123^I diagnostic scans performed according to these guidelines. The shared expertise of different medical and surgical specialties in an MDT setting facilitates discussion around optimal postoperative treatment plans in line with established protocols, and likely contributed to our finding of improved adherence to ATA guidelines. This supports the importance of an MDT in the postoperative care of this population.

## Author Contributions

**Shravan Gowrishankar**, project design; project conduct; data collection; data analysis and interpretation; manuscript writing and revision. **Dylan Thompson**, data collection; data analysis and interpretation; manuscript writing and revision. **Sara K. Bartz**, project conduct; data interpretation; manuscript writing and revision. **Lindsey S. Johnstone**, project conduct; data interpretation; manuscript writing and revision. **Kalpnaben Patel**, project conduct; manuscript writing and revision. **Chiraysu Shah**, project conduct; data interpretation; manuscript writing and revision. **Sara H. Duffus**, project conduct; data interpretation; manuscript writing and revision. **Ryan H. Belcher**, project conception; project design; project conduct; data interpretation; manuscript writing and revision.

## Disclosures

### Competing interests

The authors declare no conflicts of interest.

### Funding source

None.
